# Cytotoxic, Apoptosis-Inducing Activities, and Molecular Docking of a New Sterol from Bamboo Shoot Skin *Phyllostachys heterocycla* var. pubescens

**DOI:** 10.3390/molecules25235650

**Published:** 2020-11-30

**Authors:** Reda F. A. Abdelhameed, Mohamed S. Nafie, Ahmed K. Ibrahim, Koji Yamada, Maged S. Abdel-Kader, Amany K. Ibrahim, Safwat A. Ahmed, Jihan M. Badr, Eman S. Habib

**Affiliations:** 1 Department of Pharmacognosy, Faculty of Pharmacy, Suez Canal University, Ismailia 41522, Egypt; omarreda_70@yahoo.com (R.F.A.A.); ahmedkhider1993@gmail.com (A.K.I.); amany_mohamed@pharm.suez.edu.eg (A.K.I.); safwat_ahmed@pharm.suez.edu.eg (S.A.A.); jihanbadr2010@hotmail.com (J.M.B.); emansnd@yahoo.com (E.S.H.); 2 Department of Chemistry, Faculty of Science, Suez Canal University, Ismailia 41522, Egypt; mohamed_nafie@science.suez.edu.eg; 3 Garden for Medicinal Plants, Graduate School of Biomedical Sciences, Nagasaki University, Bunkyo-machi 1-14, Nagasaki 852-8521, Japan; kyamada@nagasaki-u.ac.jp; 4 Department of Pharmacognosy, College of Pharmacy, Prince Sattam Bin Abdulaziz University 173, Al-Kharj 11942, Saudi Arabia

**Keywords:** apoptosis, cytotoxic activity, *Phyllostachys heterocycle*, molecular docking, RT-PCR

## Abstract

Phytochemical screening of nonpolar fractions from the methanol extract of the Bamboo shoot skin *Phyllostachys heterocycla* var. pubescens resulted in the isolation of a new sterol-glucoside-fatty acid derivative (6’-*O*-octadeca-8″,11″-dienoyl)-sitosterol-3-*O*-*β*-d-glucoside (**1**), together with six known compounds. The chemical structures of the pure isolated compounds were deduced based on different spectral data. The isolated compounds were assessed to determine their cytotoxic activity, and the results were confirmed by determining their apoptotic activity. Compound **1** was more cytotoxic against the MCF-7 cells (IC_50_ = 25.8 µM) compared to Fluorouracil (5-FU) (26.98 µM), and it significantly stimulated apoptotic breast cancer cell death with 32.6-fold (16.63% compared to 0.51 for the control) at pre-G1 and G2/M-phase cell cycle arrest and blocked the progression of MCF-7 cells. Additionally, RT-PCR results further confirmed the apoptotic activity of compound **1** by the upregulation of proapoptotic genes (P53; Bax; and caspases 3, 8, and 9) and downregulation of the antiapoptotic genes (BCL2). Finally, the identified compounds, especially **1**, were found to have high binding affinity towards both tyrosine-specific protein kinase (TPK) and vascular endothelial growth factor receptor (VEGFR-2) through the molecular docking studies that highlight its mode of action.

## 1. Introduction

Bamboo is a widespread plant around the world. It comprises about 75 genera and 1250 species [1]. Bamboo young shoots are delicious and can be consumed either fresh or fermented or even canned. The shoots accumulate a considerable amount of minerals, including potassium, calcium, zinc, manganese, iron, chromium, and copper, in addition to relatively lower amounts of phosphorus and selenium [2]. Fresh shoots are reported as a good source of thiamine and niacin, besides vitamins A, B6, and E [3]. Previous studies on the medicinal activities of bamboo leaves have reported their antibacterial properties [4]. Moreover, bamboo species are commonly used in folk medicine for their antipyretic and anti-inflammatory, as well as diuretic, effects [3]. The therapeutic use of bamboo leaves for treating arteriosclerosis, hypertension, cardiovascular disease, and cancer have been also reported [5]. Additionally, antioxidant and angiotensin-converting enzyme inhibition activity were also proven [6,7]. A chemical investigation of different bamboo species reported the isolation of phenolic compounds [8,9,10] and lignans [11], besides volatile compounds [12]. Previous studies demonstrated extracts from different parts of bamboo with different chemical compositions and promising antioxidative, anticancer, antibacterial, and antiallergic activities [13,14]. Some fractionated compounds from bamboo were previously screened for their anticancer activities with apoptosis induction, among which was a porphyrin photosensitizer that was extracted from bamboo leaves, and induced apoptotic cell death in human leukemia CMK-7 and colon adenocarcinoma Colo320 DM cell lines by caspase-3 activation and DNA cleavage [15].

Tyrosine-specific protein kinase (TPK) is overexpressed in solid tumors and initiate its proliferation, so the escalation of drugs inhibiting TPK contributes as a significant insight in cancer treatment. The blocking of the vascular endothelial growth factor receptor (VEGFR-2) signaling pathway has become an appealing approach in the therapy of different cancer types [16]. Accordingly, TPK and VEGFR-2 are chosen as the biological targets for implementing the docking studies for the pure isolated compounds. These studies endeavor to demonstrate the binding affinity of these compounds inside TPK and VEGFR-2, define the important residues for the stabilization of ligands in the receptor active sites, and to find out their possible modes of action. Therefore, the current work focuses on moso bamboo shoot skins (*Phyllostachys heterocycla* var. pubescens), where the nonpolar fraction of the methanolic extract was investigated for the main chemical constituents. The chemical structures of the isolated compounds were proven, and the identified compounds were screened for their cytotoxic activity and selectivity towards cancer and normal cell lines, with apoptotic investigation for the most active compound. Additionally, the mode of action was proposed through the molecular docking studies towards TPK and VEGFR-2.

## 2. Results and Discussion

### 2.1. Structure Elucidation of the Isolated Compounds

**Figure 1:**
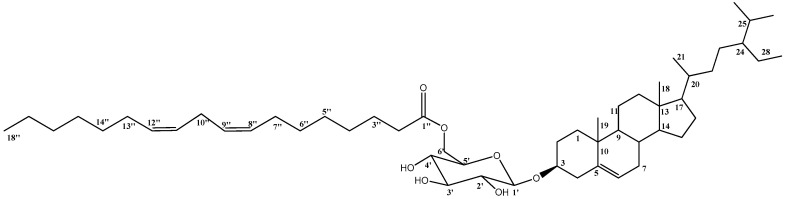
Structure of compound **1.**

**Figure 2:**
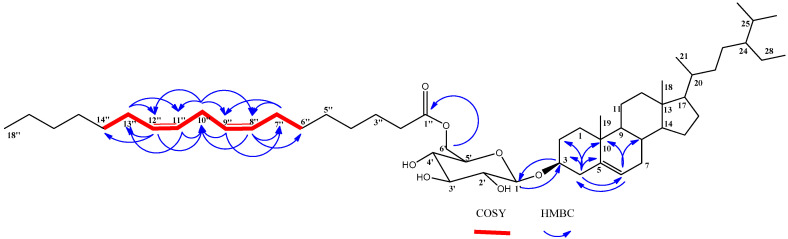
Selected HMBC and COSY correlations of compound **1.**

Compound **1** was isolated as dark-yellowish waxy material. The structure of compound **1** (Figure 1) was proven to be a sterol-glucoside-fatty acid derivative, as deduced from the combined NMR and mass spectroscopic data. Extensive examination of ^1^H NMR and ^13^C NMR spectral data, together with the data of ^1^H–^1^H COSY, DEPT, HSQC, and HMBC experiments, allowed the establishment of a C-29 steroidal skeleton with a C-5/C-6 double-bond, as indicated from the two signals resonating at δ_C_ 141.0 and 121.8, respectively. ^1^H NMR revealed a multiplet resonating at δ_H_ 3.88 attributed to H-3 with its corresponding carbon detected at δ_C_ 78.8 in the ^13^C NMR spectrum, confirming a glycoside link at this position. Combined NMR spectral data (Table 1), together with a comparison with those previously published, proved the skeleton of sitosterol-3-*O*-β-d-glucoside [17]. The remaining signals in the ^1^H NMR and ^13^C NMR spectra suggested the presence of long-chain unsaturated fatty acids with four sp^2^ carbons detected at δ_C_ 128.3, 128.4, 130.3, and 130.4, while the signal detected at δ_C_ 173.5 confirmed the presence of ester carbonyl functionality. The site of attachment of the aliphatic chain to glucose was determined to be through an ester bond at C-6′. This was based on the downfield shift of the C-6′ to δ_C_ 64.7 and the upshift of C-5′ to δ_C_ 75.0 [18]. HMBC correlation between the H_2_-6′ resonating at δ_H_ 4.76 and the signal detected at δ_C_ 173.5 assigned to the carbonyl functionality (C-1”) further supported the position of the fatty acid moiety. ^13^C NMR values of the allylic methylene carbons C-7”, C-10”, and C-13” were determined at δ_C_ 27.6, 25.9, and 27.5, respectively. This was based on the HMBC experiment, which correlated H-8”, H-9”, H-11”, and H-12” (4 H, m, δ_H_ 5.46) to C-7” (δ_C_ 27.6), C-10” (δ_C_ 25.9), and C-13” (δ_C_ 27.5). ^1^H–^1^H COSY and HSQC experiments allowed the assembly of the C-6”/C-14” unit, as indicated in Table 1 and Figure 2.

The double bonds of the unsaturated fatty ester group are assigned as Z-configurations based on the previously published reports, which proved that the ^13^C NMR chemical shifts of allylic methylene carbons <28 ppm in the case of *Z* alkenes and >30 ppm in the case of *E* ones [18,19]. Finally, to determine the exact length of the fatty acid chain, the compound was hydrolyzed, as mentioned in the experimental section, where the methylated fatty acid was subjected to GC-MS analysis. The results showed that 8,11-octadecadienoic acid-methyl ester was detected at t_R_ = 32.8 and was identified and confirmed by the library (WileyRegi stry8e and mainlib). A final confirmation of the structure was obtained from HRMS analysis (positive mode) *m/z*: 861.6581 [M+Na]^+^. Based on the previous discussion, compound **1** was assigned as the (6’-*O*-octadeca-8″,11″-dienoyl)-sitosterol-3-*O*-*β*-d-glucoside and reported here as a new natural product isolated for the first time from a natural source.

**Figure 3:**
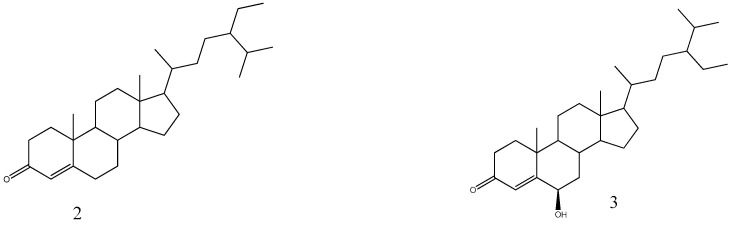

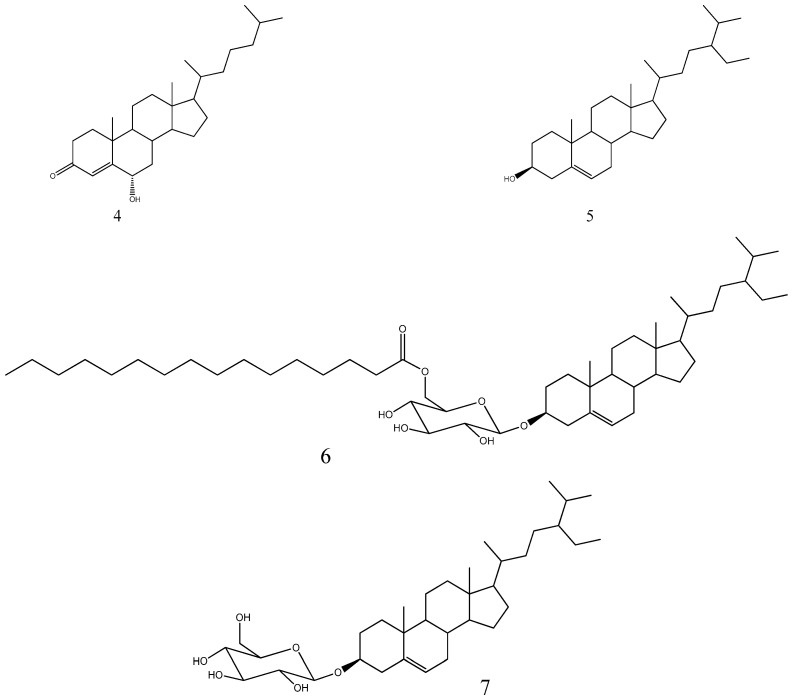
Structures of compounds **2**–**7.**

The six known compounds were identified through the analysis of the spectroscopic data, in addition to a comparison of their data with those previously presented in the literature, such as: stigmast-4-en-3-one (**2**) [20], 6-*β*-hydroxy-24-ethyl-cholest-4-en-3-one (**3**) [21], 6-α-hydroxycholest-4-en-3-one (**4**) [22], β-sitosterol (**5**) [23,24], (6’-*O*-palmitoyl)-sitosterol-3-*O*-*β*-d-glucoside (**6**) [25], and *β*-sitosterol-3-*O*-*β*-d-glucoside (**7**) [17,23]. The structures of the six compounds are illustrated in Figure 3.

### 2.2. Biological Evaluation of the Crude Extract and the Isolated Compounds

#### 2.2.1. Cytotoxic Assay

**Figure 4:**
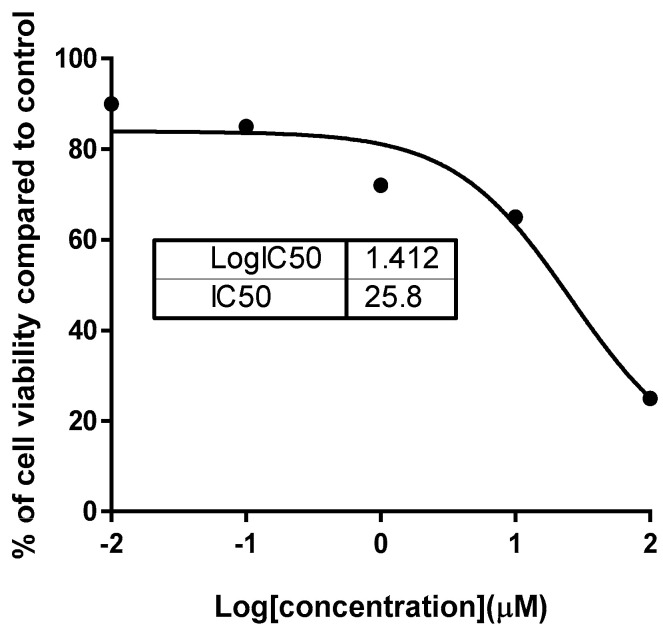
Nonlinear regression dose-inhibition curve fit of compound **1** against MCF-7 using GraphPad Prism 7 software.

Crude extracts, in addition to six sterol compounds, were screened for their cytotoxic activities against four cancerous cell lines: HepG2, Hela, A549, and MCF-7 and a noncancerous breast (MCF-10A) cell line to test their safety using the MTT assay (Table 2). The bamboo crude extract was most cytotoxic against the MCF-7 cell line (IC_50_ = 38.8 µg/mL), with cell growth inhibition 63.4% at the highest concentration of 100 µg/mL (Figure 4), while it was safe against normal cells. After fractionation and isolation of the pure compounds, six compounds were tested for their cytotoxicity. Compound **1** was cytotoxic against the MCF-7 cells (IC_50_ = 25.8 µM) ore nearly the same as the 5-fluorouracil (5-FU) 26.98 µM, and it was not toxic compared to 5-FU against the HepG2 cells (IC_50_ = 27.52 µM compared to 15.8 µM). These results indicated that it was selective in its cytotoxic activity. Additionally, it was safe against the normal cells, with IC_50_ ≥ 50. Hence, compound **1** was assumed of value to be investigated in order to determine its impact on the induction of apoptosis in MCF-7 cancer cells.

#### 2.2.2. Apoptotic Investigation

**Figure 5:**
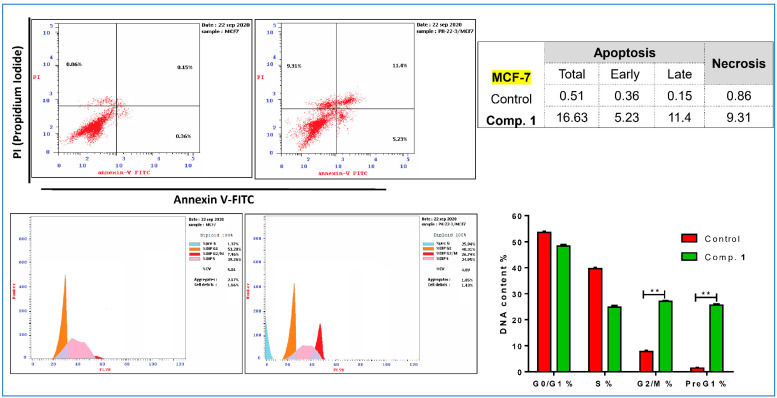
FITC/Annexin-V-FITC/PI differential apoptosis/necrosis (Upper panel) and DNA content flow cytometry-aided cell cycle analyses with bar chart representation (Lower panel) of both untreated and treated MCF-7 treated with compound **1** (IC_50_ = 25.92 μM, 48 h). ** *p* < 0.05 compared to the control.

MCF-7 cancer cells were treated with compound **1** (IC_50_ = 25.92 μM, 48 h), which was investigated for its apoptosis-inducing activity using a flow cytometric analysis of Annexin V/PI staining and cell cycle analysis with the cell population in different cell cycle phases. As seen in Figure 5, upper panel, compound **1** significantly stimulated apoptotic breast cancer cell death 32.6-fold (16.63% compared to 0.51% for the control), while it stimulated cell death via necrosis 10.8-fold (9.31% compared to 0.86% for the control). Moreover, MCF-7 cancer cells after compound **1** treatment were subjected to DNA flow cytometry to analyse the cell cycle kinetics to determine the compound’s phase interference with the cell cycle. The study of the cell cycle is a crucial test showing the cell accumulation percentage in each growth phase with cytotoxic substances after treatment. As seen in Figure 5**,** lower panel, it increased the G2/M cell (26.74% compared to 7.46% for the control) and pre-G1 (25.94% compared to 1.37% for the control) populations; additionally, it decreased the cell population in S (24.95% compared to 34.26% for the control). Consequently, compound **1** induced pre-G1 and G2/M-phase cell cycle arrest and blocked the progression of MCF-7 cancer cells that deteriorated the genetic material.

### 2.3. RT-PCR

**Figure 6:**
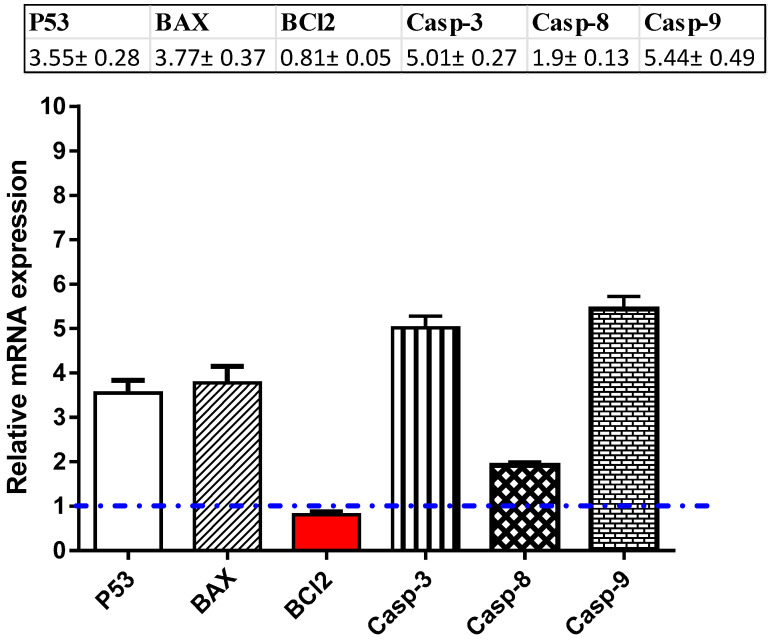
RT-PCR analysis of the apoptosis-related genes was performed after the MCF-7 cells were treated with compound **1** (IC_50_ = 25.92 μM, 48 h).

For investigation of the apoptotic pathway, MCF-7 cells were treated with compound **1** (IC_50_ = 25.92 μM, 48 h); after this RNA was extracted, cDNA was synthesized, and the RT-PCR reaction was made to follow the mRNA expression of proapoptotic P53 and BAX; caspases 3, 8, and 9; and antiapoptotic gene Bcl-2. As shown in Figure 6, compound **1** significantly activated the level of the P53 gene (≈3.55-fold), with concomitant activation of the BAX levels, with a maximum increase of 3.77-fold. The compound was able to significantly increase the mRNA levels of caspase 3, 8, and 9 genes with a maximum increase of ≈5.01-fold, 1.9-fold, and 5.44-fold, respectively, while it significantly inhibited the antiapoptotic Bcl-2 gene (maximum decrease of ≈0.81-fold). The results are in accordance with the proposed apoptotic pathway for anticancer activity.

### 2.4. Molecular Docking

**Figure 7:**
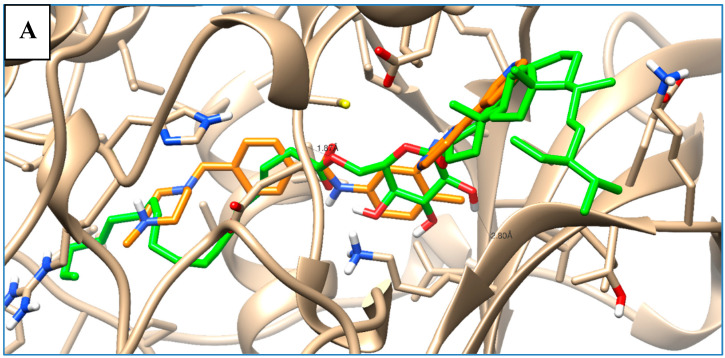

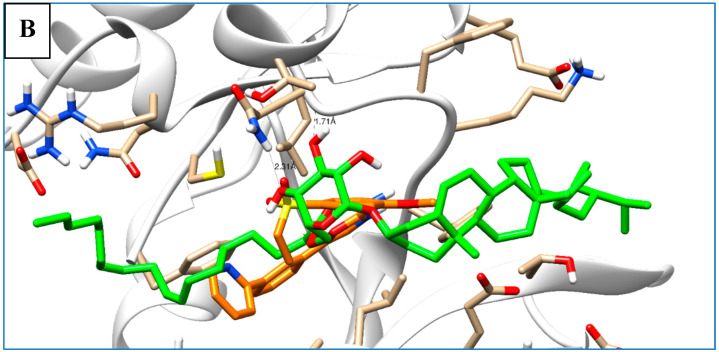
Superimposition of compound **1** (green) and the co-crystallized ligand (orange) inside both receptor-binding sites of the (**A**) tyrosine-specific protein kinase (TPK) (1T46) and (**B**) vascular endothelial growth factor receptor (VEGFR-2) (1Y6A).

Most of the examined compounds revealed significant activity against the human breast MCF-7 cancer cell line. We aimed to explore whether these compounds have a similar mechanism as the TPK and VEGFR-2 inhibitors. Accordingly, molecular docking was done to assess the binding energy, as well as the mechanism of the interaction of the compounds inside tyrosine-specific protein kinase (TPK) and vascular endothelial growth factor receptor (VEGFR-2) receptors. Compound **1** was docked inside the 1T46 protein with a binding energy of −78.58 (Kcal/mol), and it formed one hydrogen bond with a distance of 1.87 Å through its OH group as Hydrogen bond acceptor (HBA) with Lys 623 as one of the key amino acid residues of the receptor-binding site. Additionally, it was docked inside the 1Y6A protein with a binding energy of −24.56 (Kcal/mol), and it formed one hydrogen bond with a distance of 2.81 Å through its OH group as HBA with Asn 921, which is the key amino acid for the binding interaction. Therefore, these molecular docking results suggested the dual activity of the tested derivatives as protein kinase/VEGFR-2 inhibitors, and this might propose the mechanistic mode of action for their cytotoxic activities (Table 3 and Figure 7).

## 3. Experimental Section

### 3.1. General Experimental Procedures

1D and 2D NMR spectra (chemical shifts in ppm and coupling constants in Hz) were recorded on Bruker Avance DRX 500 MHz spectrometers using deuterated pyridine as the solvent for compounds **1** and **7** and CDCl_3_ as the solvent for all other compounds. Column chromatographic separations were carried out using Sephadex LH-20 (0.25–0.1 mm, Pharmacia) and silica gel 60 (0.04–0.063 mm). TLC was accomplished using precoated TLC plates with silica gel 60 F254 (0.2 mm, Merck). Spots were visualized by UV absorption at λ 255 and 366 nm, followed by spraying with P-anisaldehyde/H_2_SO_4_. High-resolution mass spectroscopy (HRMS) was determined by direct injection using Thermo Scientific UPLC RS Ultimate 3000–Q. Exactive hybrid quadrupole-Orbitrap mass spectrometer combines high-performance quadrupole precursor selection with high resolution, accurate-mass (HR/AM) Orbitrap™ detection. Detection was in both positive and negative modes separately.

### 3.2. Plant Material

Moso bamboo (*Phyllostachys heterocycla*) was obtained from Japan. The plant was harvested in Isahaya, Nagasaki, Japan and collected on October 2011 and stored at −24 °C until used. It was identified by Koji Yamada, Garden for Medicinal Plants, School of Pharmacy, Nagasaki University, Japan. A voucher specimen was deposited in the herbarium section of the Pharmacognosy Department, Faculty of Pharmacy, Suez Canal University, Ismailia, Egypt under registration number KY-11.

### 3.3. Extraction and Purification of Compounds **1**–**7**

An amount of 12.7 Kg was repeatedly extracted three times with methanol (20 L), followed by further extraction with 20 L of CHCl_3_:MeOH (1:1) at room temperature, and the combined extracts were concentrated in vacuum using a rotary evaporator to give a residue of 110 g. The residue was suspended in H_2_O (4 L) and extracted with *n*-hexane, EtOAc, and *n*-BuOH, successively. The *n*-hexane extract (37 g) was subjected to a SiO_2_ column and eluted using *n*-hexane:CHCl_3_:MeOH gradient. Three main fractions were investigated (fractions A, B, and C). Fraction A was further chromatographed to yield three main subfractions (x, xx, and xxx). The subfraction x was purified on silica gel column using isocratic elution with EtOAc:*n*-hexane (1:40), followed by purification on a Sephadex LH-20 column and eluted with CHCl_3_:MeOH (1:1) to yield a pure white powder of compound **2** (5.8 mg). The subfraction xx was purified over a Sephadex LH-20 column and eluted with CHCl_3_:MeOH (1:1), then, finally, purified on preparative TLC using EtOAc:*n*-hexane (1:3) to yield a pure white powder of compound **3** (9.4 mg). The subfraction xxx was chromatographed over a SiO_2_ column, then over a Sephadex LH-20 column, and, finally, purified using preparative TLC using EtOAc:*n*-hexane (1:1) to yield a pure white powder of compound **4** (0.3 mg). Fraction B was subjected to a SiO_2_ column then over a Sephadex LH-20 column and eluted with CHCl_3_:MeOH (1:1). Final purification was accomplished by recrystallization in CHCl_3_:MeOH (1:1) to yield pure crystalline needles of compound **5** (23.9 mg). Fraction C was fractionated over a SiO_2_ column using the MeOH:CHCl_3_ gradient, then, finally, purified by crystallization in CHCl_3_:MeOH (1:1) to yield the pure dark-yellowish waxy residue of compound **1** (310.3 mg). The EtOAc extract was subjected to a SiO2 column and eluted with the CHCl_3_:MeOH gradient. Two main fractions (D and E) were obtained. Fraction D was further chromatographed on a Sephadex LH-20 using CHCl_3_:MeOH (1:1) as the eluent. Final purification was carried out by recrystallization in CHCl_3_:MeOH (1:1) to afford the pure white powder of compound **6** (31 mg). Fraction E was chromatographed over a Sephadex LH-20 column and eluted with CHCl_3_:MeOH (1:1), then purified by recrystallization in CHCl_3_:MeOH (1:1) to yield the pure white powder of compound **7** (4.9 mg).

### 3.4. Hydrolysis of Compound **1**

Compound (**1**) was hydrolyzed, and the free fatty acid was methylated by dissolving the compound in toluene (1.2 mL), methanol (1.5 mL), and then, 0.3 mL of 8% HCl solution was added; the final solution was incubated at 45°C overnight and, then, the aqueous solution was fractionated against *n*-hexane, and methylated fatty acid was extracted in the organic layer and subjected to GC-MS analysis [26].

### 3.5. Spectroscopic Data of the Isolated Compounds

*6’-O-octadeca-8″,11″-dienoyl)-sitosterol-3-O-β-D-glucoside* (**1**): dark-yellowish waxy residue. LC-HRMS analysis (positive mode) *m/z*: 861.6581 [M + Na]^+^ , molecular formula: C_53_H_90_O_7_, NMR data: see Table 1.

*Stigmast-4-en-3-one* (**2**): white powder; ^13^C-NMR (125 MHz, CDCl_3_): δ_C_ 35.7 (C-1), 33.9 (C-2), 199.7 (C-3), 123.7 (C-4), 171.7 (C-5), 32.9 (C-6), 32.1 (C-7), 36.1 (C-8), 53.8 (C-9), 38.6 (C-10), 21.0 (C-11), 39.6 (C-12), 42.4 (C-13), 55.9 (C-14), 24.2 (C-15), 28.2 (C-16), 56.0 (C-17), 11.9 (C-18), 17.4 (C-19), 35.6 (C-20), 18.7 (C-21), 34.0 (C-22), 26.0 (C-23), 45.8 (C-24), 29.1 (C-25), 19.8 (C-26), 19.0 (C-27), 23.1 (C-28), 11.95(C-29).

*6-Hydroxy-24-ethyl-cholest-4-en-3-one* (**3**): white powder; ^13^C-NMR (125 MHz, CDCl_3_): δ_C_ 37.1 (C-1), 34.3 (C-2), 200.6 (C-3), 126.3 (C-4), 168.6 (C-5), 73.3 (C-6), 38.6 (C-7), 29.7 (C-8), 53.6 (C-9), 38.0 (C-10), 21.0 (C-11), 39.6 (C-12), 42.5 (C-13), 56.1 (C-14), 24.1 (C-15), 28.2 (C-16), 55.9 (C-17), 12.0 (C-18), 19.5 (C-19), 36.1 (C-20), 18.7 (C-21), 33.9 (C-22), 26.1 (C-23), 45.8 (C-24), 29.1 (C-25), 19.8 (C-26), 19.0 (C-27), 23.1 (C-28), 12.0 (C-29).

*6-Hydroxycholest-4-en-3-one* (**4**): white powder; ^13^C-NMR (125 MHz, CDCl_3_): δ_C_ 36.3 (C-1), 33.8 (C-2), 199.5 (C-3), 119.6 (C-4), 171.6 (C-5), 68.7 (C-6), 41.5 (C-7), 29.7 (C-8), 53.8 (C-9), 39.1 (C-10), 19.8 (C-11), 42.5 (C-12), 45.8 (C-13), 55.6 (C-14), 29.1 (C-15), 26.0 (C-16), 55.9 (C-17), 12.0 (C-18), 19.0 (C-19), 34.2 (C-20), 18.3 (C-21), 36.1 (C-22), 24.2 (C-23), 28.1 (C-24), 39.4 (C-25), 23.1 (C-26), 21.0 (C-27).

β-sitosterol (**5**): colorless needle crystals; ^13^C-NMR (125 MHz, CDCl_3_): δ_C_ 37.3 (C-1), 31.9 (C-2), 71.8 (C-3), 42.3 (C-4), 140.8 (C-5), 121.7 (C-6), 31.6 (C-7), 31.9 C-8), 50.1 (C-9), 36.5 (C-10), 21.1 (C-11), 39.8 (C-12), 42.3 (C-13), 56.8 (C-14), 24.3 (C-15), 28.3 (C-16), 56.1 (C-17), 11.9 (C-18), 19.1 (C-19), 36.2 (C-20), 18.8 (C-21), 33.9 (C-22), 26.1 (C-23), 45.8 (C-24), 29.1 (C-25), 19.8 (C-26), 19.4 (C-27), 23.1 (C-28), 12.0 (C-29).

*(6’-O-palmitoyl)-sitosterol-3-O-β-*d*-glucoside* (**6**): white powder; ^13^C-NMR (125 MHz, CDCl_3_): δ_C_ 37.3 (C-1), 29.5 (C-2), 79.7 (C-3), 39.7 (C-4), 140.3 (C-5), 122.0 (C-6), 31.8 (C-7), 31.9 (C-8), 50.1 (C-9), 36.1 (C-10), 21.0 (C-11), 38.8 (C-12), 42.3 (C-13), 56.7 (C-14), 24.3 (C-15), 28.2 (C-16), 56.1 (C-17), 11.8 (C-18), 19.0 (C-19), 36.6 (C-20), 18.7 (C-21), 33.9 (C-22), 29.1 (C-23), 45.7 (C-24), 26.1 (C-25), 19.3 (C-26), 19.7 (C-27), 23.0 (C-28), 11.9 (C-29), 101.2 (C-1′), 73.6 (C-2′), 76.3 (C-3′), 70.5 (C-4′), 73.2 (C-5′), 63.9 (C-6′), 174.2 (C-1″), 34.3 (C-2″), 25.0 (C-3″), 29.3–29.9 (C-4″: C-14″), 22.6 (C-15″), 14.1 (C-16″).

β-sitosterol-3-*O*-β-d-glucoside (**7**): white powder; ^13^C-NMR (125 MHz, C_5_D_5_N): δ_C_ (37.3) C-1, 30.0 (C-2), 78.2 (C-3), 39.7 (C-4), 140.7 (C-5), 121.7 (C-6), 32.0 (C-7), 31.8 (C-8), 50.1 (C-9), 36.7 (C-10), 21.1 (C-1), 39.1 (C-12), 42.3 (C-13), 56.6 (C-14), 24.3 (C-15), 28.4 (C-16), 56.0 (C-17), 11.8 (C-18), 19.2 (C-19), 36.2 (C-20), 18.8 (C-21), 34.0 (C-22), 26.1 (C-23), 45.8 (C-24), 29.2 (C-25), 19.8 (C-26), 19.0 (C-27), 23.2 (C-28), 12.0 (C-29), 102.3 (C-1′), 75.1 (C-2′), 78.3 (C-3′), 71.4 (C-4′), 78.0 (C-5′), 62.5 (C-6′).

### 3.6. Biological Evaluation of the Compounds

#### 3.6.1. Cytotoxic Activity

Crude extract, together with six of the identified sterols, were screened for their cytotoxic activities against liver HepG2, breast MCF-7, and normal cells (MCF-10A), as well. According to the standard cell culture work [27,28], each cell line was cultured in a proper complete medium. The treatment of cells was performed with the crude extract and then, with the identified compounds for a 48-h incubation, and Fluorouracil (5-FU) was used as the standard. Percent of cell viability was calculated by the following formula: % cell viability = (Mean absorbance of tested compound)/(Mean absorbance in control) × 100. Then, IC_50_ in µM was calculated using GraphPad Prism 7.

#### 3.6.2. Apoptotic Investigation Using Flow Cytometric Analysis

Cell cycle analysis, as well as apoptotic assays, were performed in detail according to the previously described methods [29,30,31]. Full methodologies of flow cytometric assays were provided in the Supplementary File. After treatment with compound **1** (IC_50_ = 25.92 μM, 48 h), the harvested MCF-7 cells were subjected to flow cytometric analyses, including FITC/Annexin-V-FITC/PI differential apoptosis/necrosis assessment, and DNA content flow cytometry-aided cell cycle analysis, to define in which phase cells would be arrested and to estimate the percentage of apoptotic cells.

#### 3.6.3. RT-PCR Assay

MCF-7 cells were treated with compound **1** (IC_50_ = 25.92 μM, 48 h). At the end of treatments, cells were collected, and total RNA was extracted using a RNeasy^®^ Mini Kit (Qiagen, Hilden, Germany) according to the manufacturer’s instructions. cDNA synthesis was performed with 500 ng of RNA using the i-Script cDNA synthesis kit (Bio-Rad, Hercules, CA, USA) following the manufacturer’s instructions. Real-time (RT)-PCR reactions consisted of 25-µL Fluocycle^®^II SYBR^®^ (Euroclone, Milan, Italy), 1.5 µL of both 10-µM forward and reverse primers, 3-µL cDNA, and 19 µL of H_2_O. All reactions were performed for 35 cycles using this temperature profile: 95 °C for 5 m (initial denaturation); 95 °C for 15 min (Denaturation), 55 °C for 30 min (Annealing), and 72 °C for 30 min (Extension) [32,33]. Then, The cycle threshold (Ct) values were collected and the relative folds of changes between all the samples. Primer used were listed in the Supplementary Files.

### 3.7. In Silico Molecular Docking

As a trial to elucidate the cytotoxic activity profile exhibited by the compounds under investigation, molecular docking was performed between the studied compounds as ligands with tyrosine-specific protein kinase (TPK; PD: 1T46) and vascular endothelial growth factor receptor (VEGFR-2; PD:1Y6A). The molecular modeling studies were implemented using a computational software basis (MOE 2008-10, Chemical Computing Group, Montreal, QC, Canada), as regards the tested proteins whose crystal structures complexed with their co-crystallized ligands were easily accessible from the Protein Data bank. Basis of modeling concerning receptor and ligand preparation and molecular docking were achieved according to Nafie et al., 2019 [34,35]. Each ligand-receptor complex was examined for the binding interaction analysis.

## 4. Conclusions

Phytochemical screening of nonpolar extracts of the bamboo shoot skin *Phyllostachys heterocycla* resulted in the isolation of the new compound (6’-*O*-octadeca-8″, 11″-dienoyl)-sitosterol-3-*O*-*β*-d-glucoside, together with six known sterols. The new compound showed potent cytotoxic effects against the MCF-7 cells (IC_50_ = 25.8 µM). It significantly stimulated apoptotic breast cancer cell death 32.6-fold (16.63% compared to 0.51% for the control) at the pre-G1 and G2/M-phase cell cycle arrest and blocked the progression of MCF-7 cells. Moreover, the RT-PCR results further confirmed the apoptotic activity of compound **1** by the upregulation of proapoptotic genes (P53; Bax; caspases 3, 8, and 9) and downregulation of the antiapoptotic genes (BCL2). Finally, molecular docking studies could declare the mode of action of the tested compounds, as they were found to have a high binding affinity towards both TPK and VEGFR-2, where the new compound **1** possessed the highest affinity.

## Figures and Tables

**Table 1:** NMR spectroscopic data of **1** (C_5_D_5_N, 500 and 125 MHz).

Position	δ_C_ (m) ^a^	δ_H_ (m, *J* in Hz)	Selected HMBC ^b^
1	37.7, CH_2_	1.09, 1.78	C-19
2	32.1, CH_2_	1.44 *	
3	78.8, CH	3.88, m	C-1’
4	39.3, CH_2_	2.64, dd, *J* (12,4), 2.43, brt, *J* (8)	C-2, C-3, C-5, C-6, C-10
5	141.0, C		
6	121.8, CH	5.37, m	C-4, C-8, C-10
7	31.7, CH_2_	1.48, 1.91	C-5
8	32.1, CH	1.27	C-9, C-11, C-14
9	50.5, CH	0.94 **	C-1, C-5
10	36.9 C		
11	21.4, CH_2_	1.44 *	
12	40.1, CH_2_	2.05, 1.27	
13	42.5 C		
14	56.9, CH	1.01, m	
15	24.6, CH_2_	1.09, 1.61	
16	28.6, CH_2_	2.12, m	
17	56.3, CH	1.15	
18	12.0, CH_3_	0.72, s	C-12, C-17
19	19.3, CH_3_	0.94, s	C-1, C-5
20	36.5, CH	1.44 *	
21	19.1, CH_3_	1.01, d, *J* (6.0)	
22	34.2, CH_2_	1.09, 1.42	C-17, C-21
23	26.4, CH_2_	1.27	
24	46.1, CH	1.01	
25	29.5, CH	1.27 ***	
26	19.4, CH_3_	0.87 ****	
27	20.0, CH_3_	0.90 **	
28	23.4, CH_2_	1.29 ***	
29	12.2, CH_3_	0.88 ****	
1’	102.7, CH	4.91, d, *J* (7.2)	C-3, C-3’
2’	75.0, CH	3.97, d, *J* (8.1)	C-1’, C-3’
3’	78.2, CH	4.17, m	C-4’
4’	71.6, CH	3.97, m	C-3’
5’	75.0, CH	3.96, m	C-1’, C-3’
6’	64.7, CH_2_	4.76, m	C-1″
1″	173.5 C		
2″	34.5, CH_2_	2.37, m	C-1″, C-3″, C-4″
3″	25.4, CH_2_	1.65, m	C-1″, C-2″, C-4″, C-5″
4″	29.5–32.1, CH_2_	1.32, m	
5″	29.5–32.1, CH_2_	1.32, m	
6″	29.5–32.1, CH_2_	1.32, m	
7″	27.6, CH_2_	2.12, m	C-5″, C-6″, C-8″, C-9″
8″	128.3, CH	5.46, m	C-6″, C-7″, C-10″
9″	128.4, CH	5.46, m	C-7″, C-10″
10″	25.9, CH_2_	2.93, t, *J* (6.4)	C-8″, C-9″, C-11″, C-12″
11″	130.3, CH	5.46, m	C-10″, C-13″
12″	130.4, CH	5.46, m	C-10″, C13″, C-14″
13″	27.5, CH_2_	2.12, m	C-11″, C-12″, C-14″, C-15″
14″	29.5–32.1, CH_2_	1.32, m	
15″	29.5–32.1, CH_2_	1.32, m	
16″	29.5–32.1, CH_2_	1.32, m	
17″	29.5–32.1, CH_2_	1.32, m	
18″	14.3, CH_3_	0.87 ****	

^a^ Multiplicities were deduced from multiplicity-edited HSQC. ^b^ HMBC correlations are from proton(s) stated to the indicated carbons. *, **, ***, and ****: overlapped signals.

**Table 2:** Summarized IC_50_ of crude and identified sterol derivatives against four cancerous cell lines: HepG2, Hela, A549, and MCF-7 and normal cells.

Tested Samples	IC_50_ ± SD (µg/mL) *^,#^				
	HepG2	Hela	A549	MCF-7	Normal Cells MCF-10A
**Crude Extract**	48.4 ± 1.32	≥50	ND	38.87 ± 0.87	≥50
**Pure Compounds**	IC_50_ ± SD (µM) *^,#^				
**1**	27.52 ± 1.04	ND	ND	25.82 ± 1.04	≥50
**2**	ND	32.45 ± 0.89	45.63 ± 0.75	≥50	
**3**	ND	≥50	49.9 ± 1.03	≥50	
**5**	≥50	42.32 ± 1.02	≥50	≥50	
**6**	42.46 ± 1.71	ND	ND	≥50	
**7**	≥50	ND	ND	38.05 ± 0.98	
5-FU	15.8 ± 0.28	13.04 ± 0.65	7.47 ± 0.43	26.98 ± 0.76	

* Values are expressed as mean ± SD of 3 independent trials (n = 3). ND is nondetermined. **^#^** IC_50_ were calculated using GraphPad Prism 7 software using a nonlinear regression dose-inhibition curve fit. 5-FU: 5- fluorouracil.

**Table 3:** Ligand receptor interactions of compound **1** ^#^ inside the tyrosine-specific protein kinase (TPK) (1T46) and vascular endothelial growth factor receptor (VEGFR-2) (1Y6A) targets. PDB: Protein Data Bank.

Molecular Target and PDB Code	Binding Energy (Kcal/Mol)	Moiety of Compound	Distance (A)	Key Amino Acid Residues *
Protein kinase (TPK) (1T46)	−78.58	HO- (HBA)	1.87	Lys 623
VEGFR-2 (1Y6A)	−24.56	HO- (HBA)	2.80	Asn 921
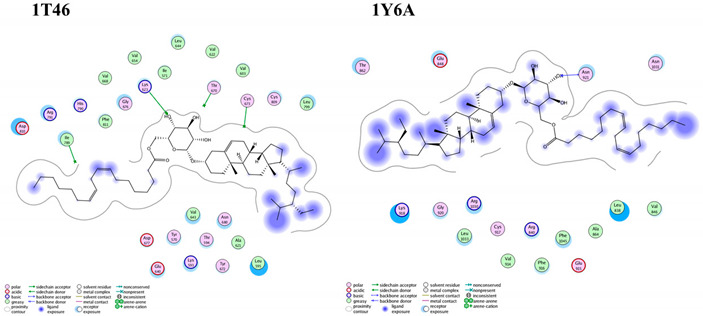				

* Amino acids with which the co-crystallized ligand interacts inside the receptor-binding site. ^#^ Docking results of the rest of the compounds were supported as supplemental.

## Supplementary Materials

The following are available online. Full characterization charts (^1^HNMR, ^13^CNMR, DEPT-135, COSY, HMBC, HSQ C, and mass spectroscopy) of the new compound, molecular docking files, and results are supported as a Supplementary File.
