# Supplementary material for: Cytotoxic, Apoptosis-Inducing Activities, and Molecular Docking of a New Sterol from Bamboo Shoot Skin Phyllostachys heterocycla var. pubescens
Source: Molecules. 2020 Nov 30;25(23):5650. doi: 10.3390/molecules25235650 (PMC7731115; doi:10.3390/molecules25235650)
Supplement: Supplementary file 1 [file molecules-25-05650-s001.zip › Electronic supplementry Material (ESM)/Codes-for compounds.docx]

**Electronic supplementary material**

Full characterization charts (^1^HNMR, ^13^CNMR, DEPT135, COSY, HMBC, HSQC, and Mass spectroscopy), molecular docking files and results are supported as supplementary file.

**Codes of the compounds**

Compound **1** PH-H22-3

Compound **2** PH-H12-2-4

Compound **3** PH-H12-7

Compound **4** PH-H12-8-4

Compound **5** PH-H16-1

Compound **6** PH-E14-12

Compound **7** PH-E14-16
