# Supplementary material for: Cytotoxic, Apoptosis-Inducing Activities, and Molecular Docking of a New Sterol from Bamboo Shoot Skin Phyllostachys heterocycla var. pubescens
Source: Molecules. 2020 Nov 30;25(23):5650. doi: 10.3390/molecules25235650 (PMC7731115; doi:10.3390/molecules25235650)
Supplement: Supplementary file 1 [file molecules-25-05650-s001.zip › Electronic supplementry Material (ESM)/spectral data supp. file .pdf]

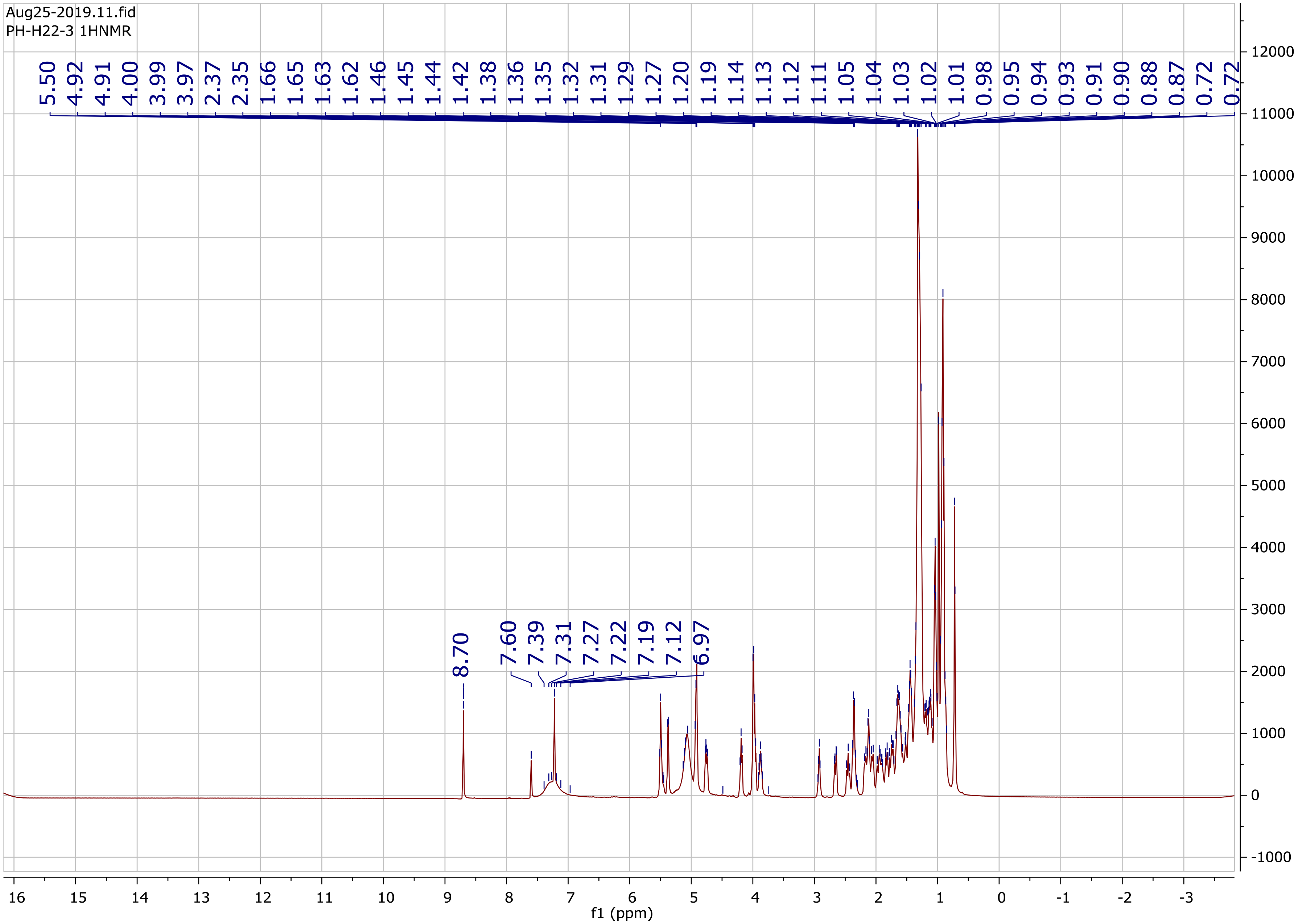

Figure 1: ^{1}HNMR spectrum of compound 1

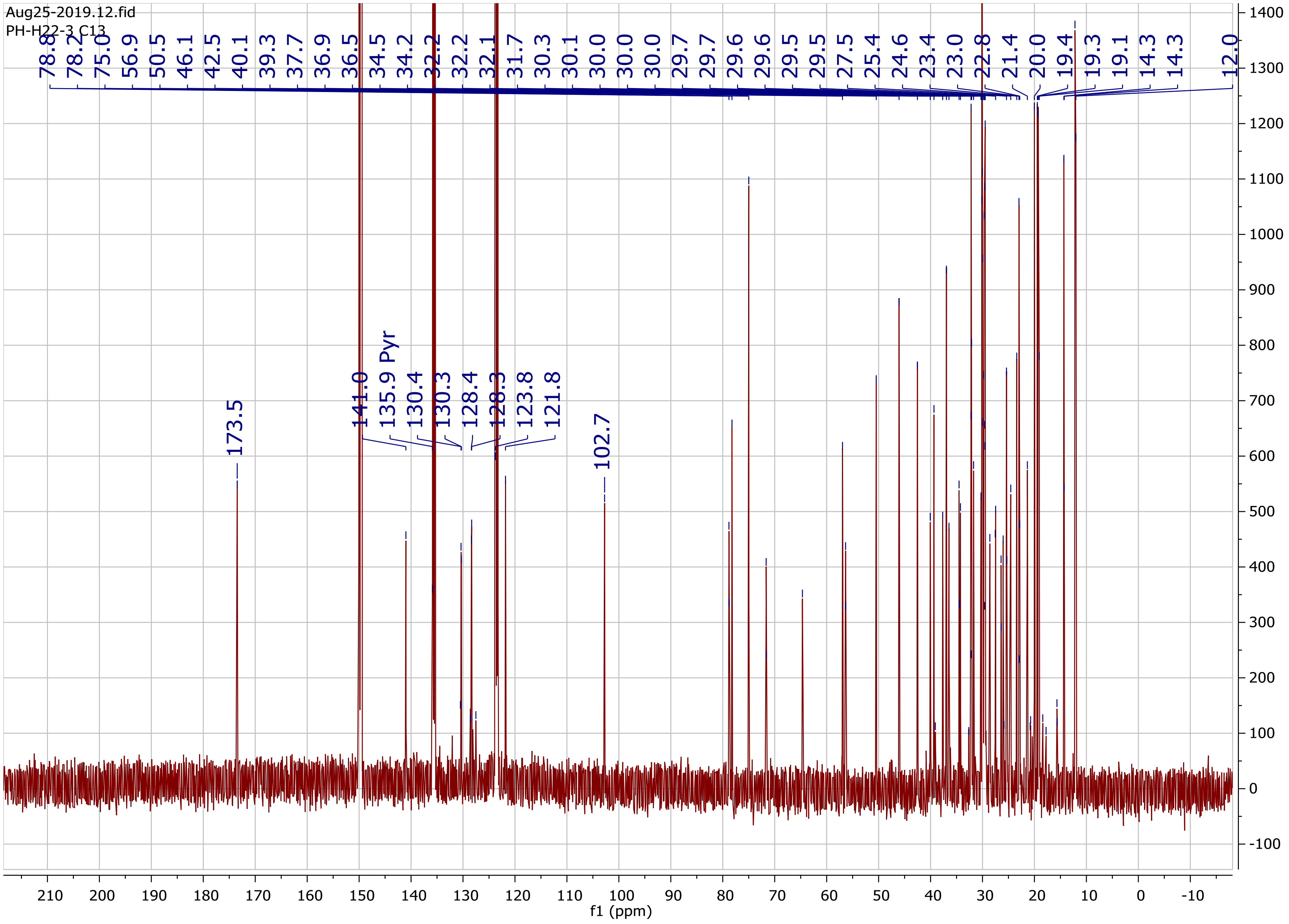

Figure 2: ^{13}CNMR spectrum of compound 1

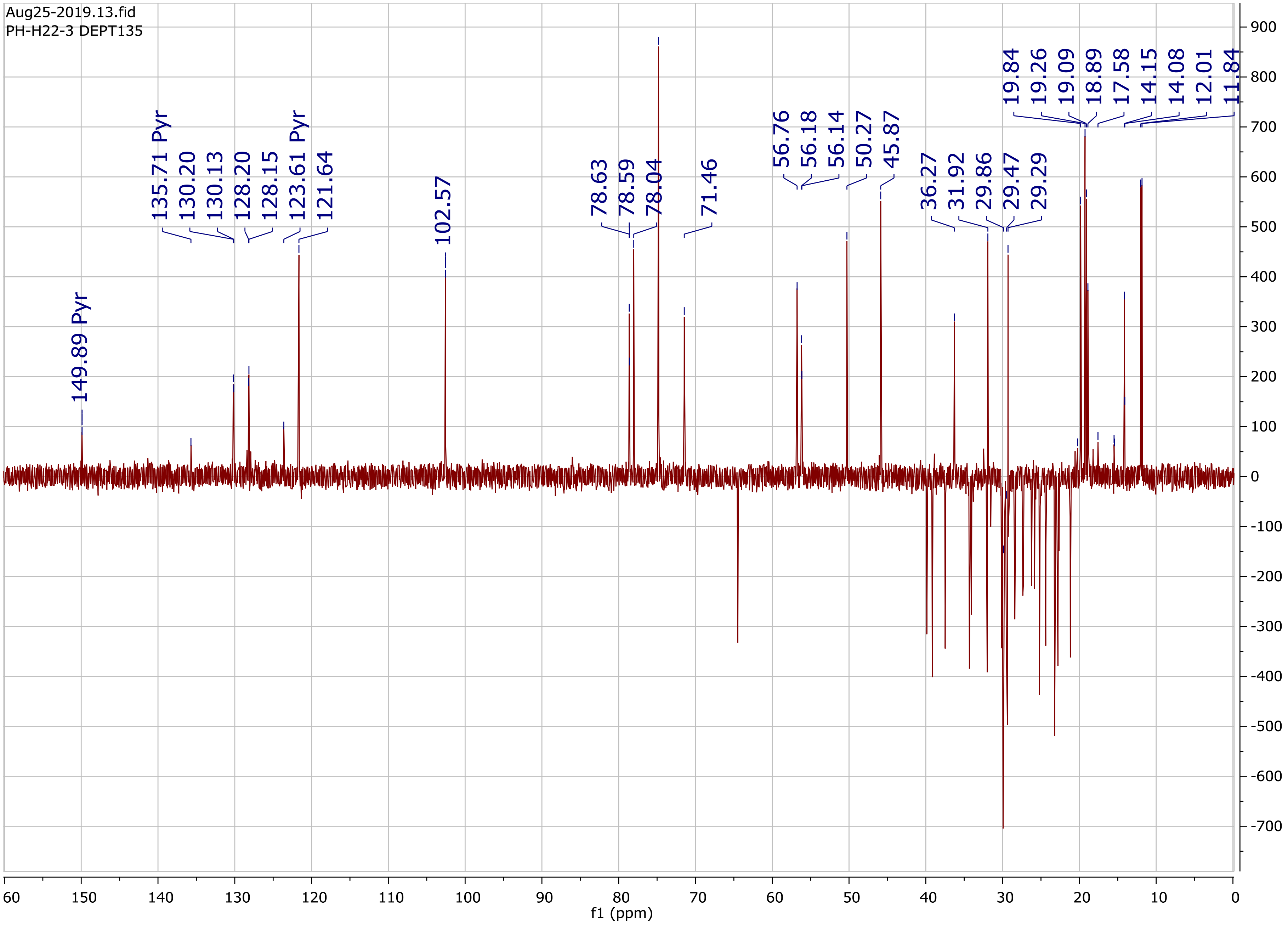

Figure 3: DEPT135 spectrum of compound 1

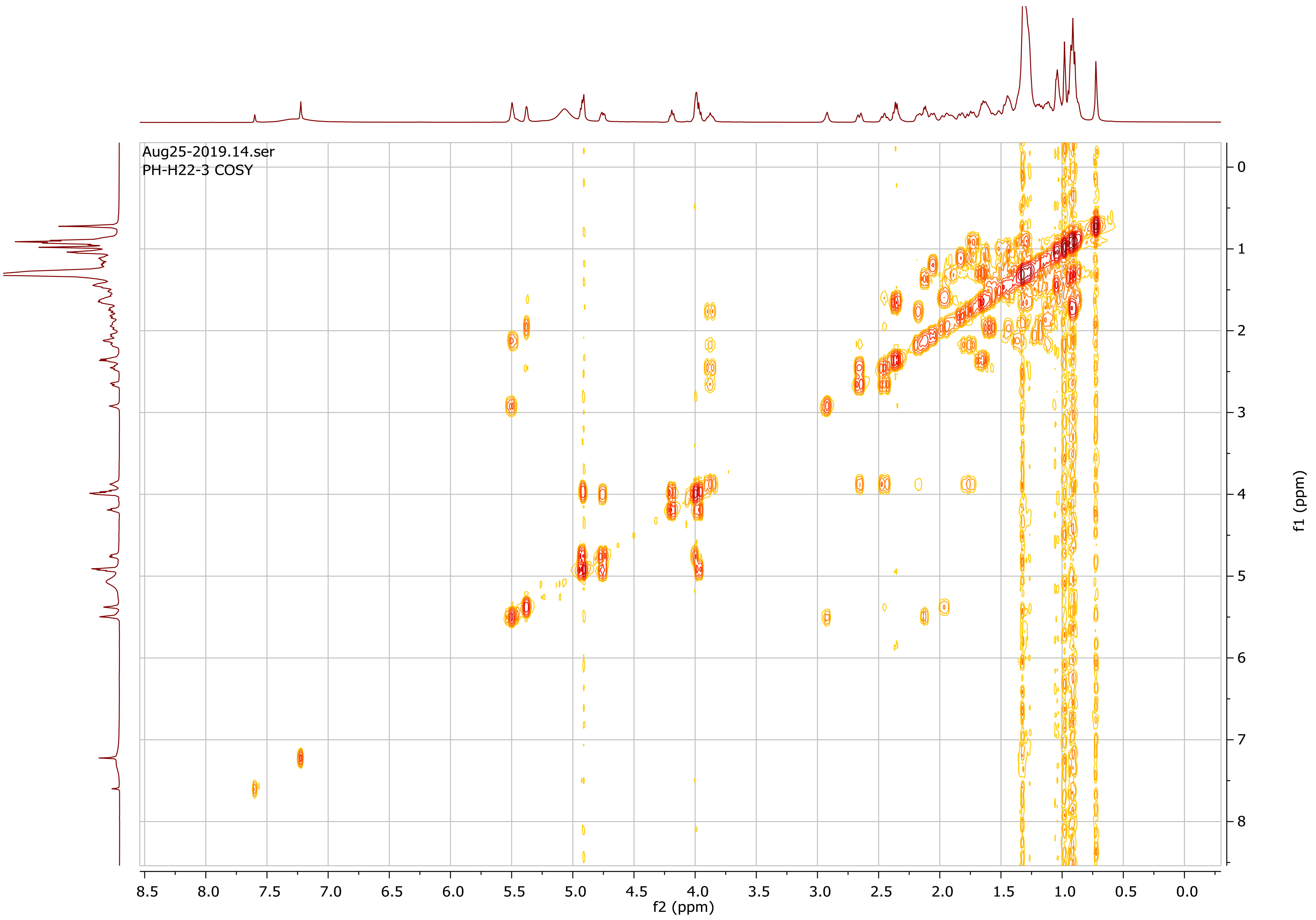

Figure 4: COSY spectrum of compound 1

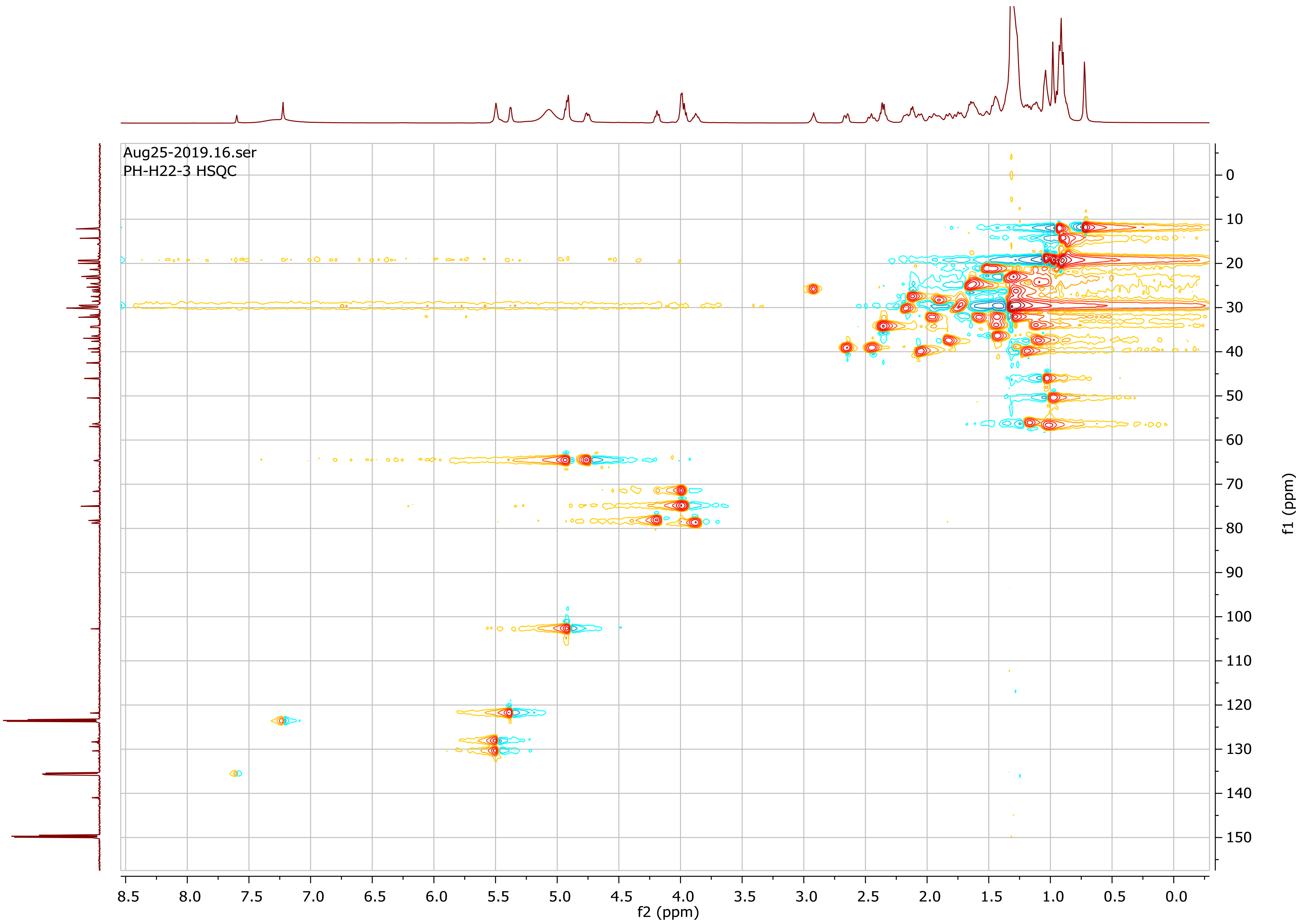

Figure 5: HSQC spectrum of compound 1

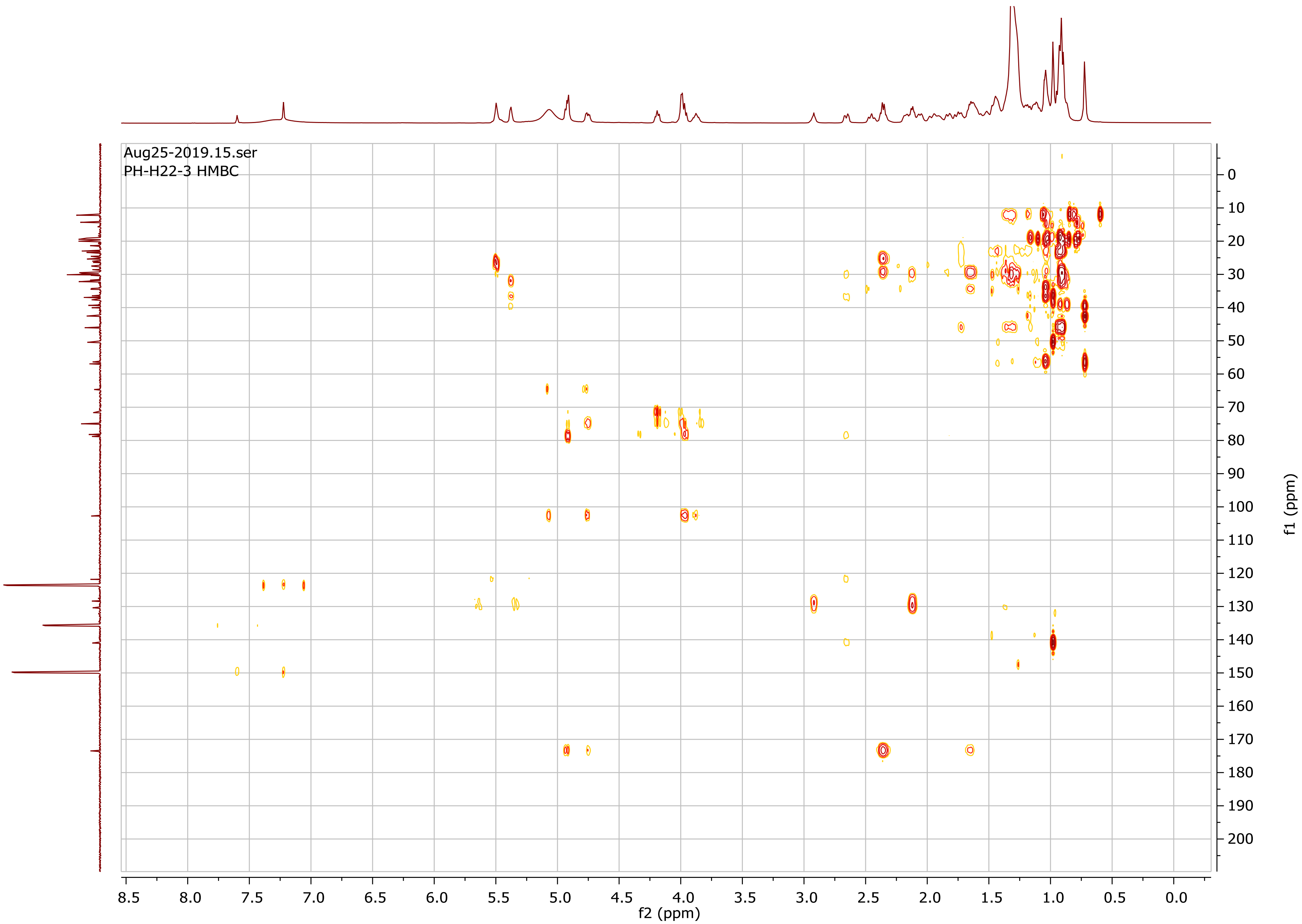

Figure 6: HMBC spectrum of compound 1

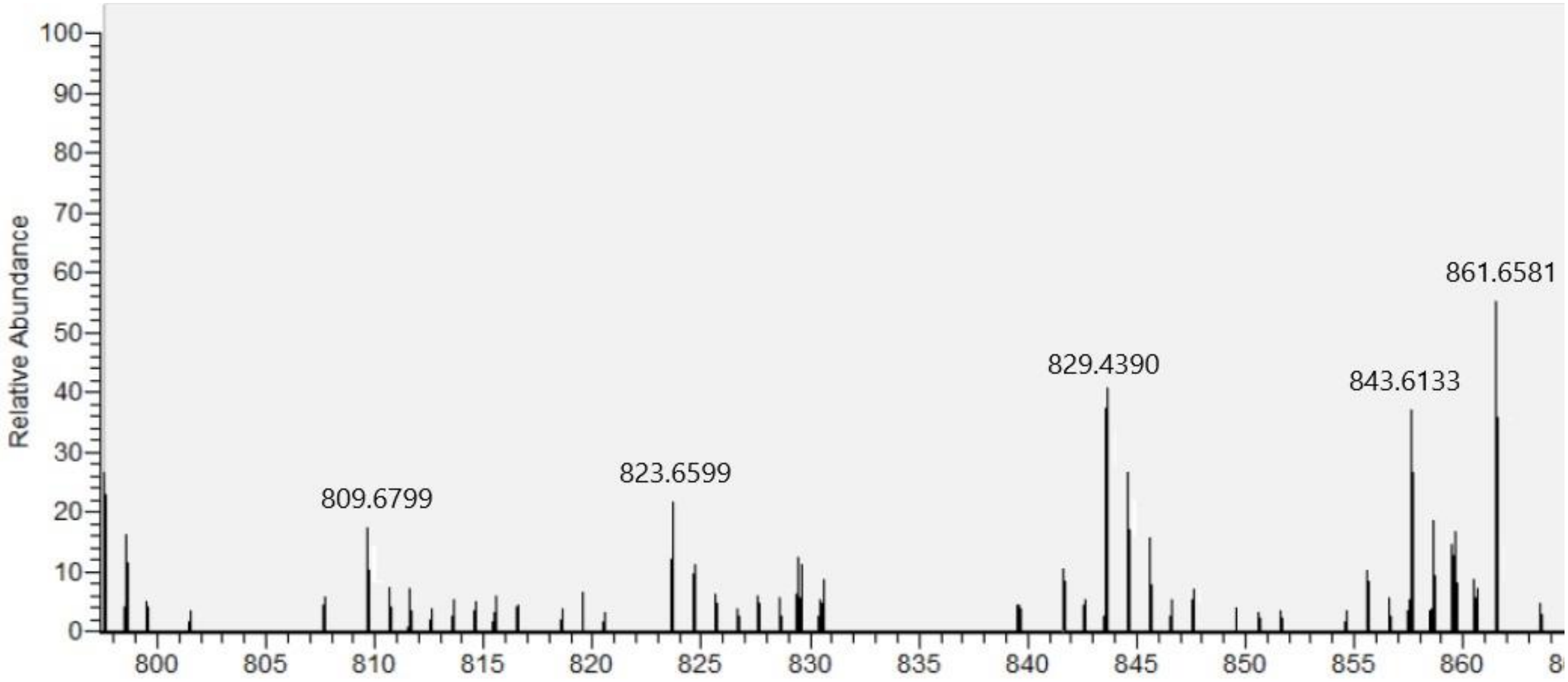

Figure 7: Mass spectrum of compound 1
