# Supplementary material for: Cytotoxic, Apoptosis-Inducing Activities, and Molecular Docking of a New Sterol from Bamboo Shoot Skin Phyllostachys heterocycla var. pubescens
Source: Molecules. 2020 Nov 30;25(23):5650. doi: 10.3390/molecules25235650 (PMC7731115; doi:10.3390/molecules25235650)
Supplement: Supplementary file 1 [file molecules-25-05650-s001.zip › Electronic supplementry Material (ESM)/supplementy data of sterols of Phyllostachys heterocycle-All (New+Known).docx]

**A New Sterol with Potent Apoptotic Effect from Bamboo Shoot**

**Skin *Phyllostachys heterocycle***

Reda F. A. Abdelhameed^1^, Mohamed S. Nafie^2^, Ahmed K. Ibrahim^1^, Koji Yamada^3^, Maged S. Abdel-Kader^4*^, Amany K. Ibrahim^1^, Safwat A. Ahmed^1^, Jihan M. Badr^1^, Eman S. Habib^1^

^1^Department of Pharmacognosy, Faculty of Pharmacy, Suez Canal University, Ismailia 41522, Egypt

^2^Department of Chemistry, Faculty of Science, Suez Canal University, Ismailia 41522, Egypt

^3^Garden for Medicinal Plants, Graduate School of Biomedical Sciences, Nagasaki University; Bunkyo-machi 1-14, Nagasaki 852-8521, Japan

^4^Department of Pharmacognosy, College of Pharmacy, Prince Sattam Bin Abdulaziz University, 173, AlKharj 11942, Saudi Arabia.

*Corresponding author:

Maged S. Abdel-Kader

Tel. : +966545539145

Email: mpharm101@hotmail.com

**Table of Contents**

**Figure S1:** ^1^H-NMR spectrum of compound 1 (500 MHz, C_5_D_5_N**)**…………………………………....…......5

**Figure S2:** ^13^C-NMR spectra of compound 1 **(**125 MHz, C_5_D_5_N**)**…...…………….…………...……..….…..6

**Figure S3:** DEPT135 of compound 1…………...………..…….…..…..........................….…………..……...7

**Figure S4:** COSY of compound 1………..….…..............................................................................................8

**Figure S5:** HMPC of compound 1………………..……...……………….......................................................9

**Figure S6:** HSQC of compound 1…………………….….….………....……………......................................10

**Figure S7:** LC-HRMS analysis (positive mode) [M+Na] + of compound 1 …………....…………....…........11

**Figure S8:** GC-MS of the fatty acid liberated from compound 1 after hydrolysis …….….….……...............12

**Figure S9:** ^1^H-NMR spectrum of compound 2 (500 MHz, CDCl_3_**)**……………………………………..........13

**Figure S10:** ^13^C-NMR spectra of compound 2 **(**125 MHz, CDCl_3_**)**…….….….……...………..….….............14

**Figure S11:** DEPT135 of compound 2…………………..………………....…...............................................15

**Figure S12:** COSY of compound 2………………….….….……....………..….…........................................16

**Figure S13:** HMPC of compound 2……………………………………....…..................................................17

**Figure S14:** HSQC of compound 2………………….….….……...………..….….........................................18

**Figure S15:** ^1^H-NMR spectrum of compound 3 (500 MHz, CDCl_3_**)**…………………………………….......19

**Figure S16:** ^13^C-NMR spectra of compound 3 **(**125 MHz, CDCl_3_**)**…….….….……...………..….…............20

**Figure S17:** DEPT135 of compound 3…………………..………………....…...............................................21

**Figure S18:** COSY of compound 3………………….….….……....………..….…........................................22

**Figure S19:** HMPC of compound 3……………………………………....……………………………….....23

**Figure S20:** HSQC of compound 3………………….….….……...………..….….........................................24

**Figure S21:** ^1^H-NMR spectrum of compound 4 (500 MHz, CDCl_3_**)**…………………………………….......25

**Figure S22:** ^13^C-NMR spectra of compound 4 **(**125 MHz, CDCl_3_**)**…….….….……...………..….…............26

**Figure S23:** DEPT135 of compound 4…………………..………………....…...............................................27

**Figure S24:** COSY of compound 4………………….….….……....………..….…........................................28

**Figure S25:** HMPC of compound 4……………………………………....….................................................29

**Figure S26:** HSQC of compound 4………………….….….……...………..….….........................................30

**Figure S27:** ^1^H-NMR spectrum of compound 5 (500 MHz, CDCl_3_**)**…………………………………….......31

**Figure S28:** ^13^C-NMR spectra of compound 5 **(**125 MHz, CDCl_3_**)**…….….….……...………..….…............32

**Figure S29:** DEPT135 of compound 5…………………..………………....…...............................................33

**Figure S30:** COSY of compound 5………………….….….……....………..….…........................................34

**Figure S31:** HMPC of compound 5……………………………………....……………………………….....35

**Figure S32:** HSQC of compound 5………………….….….……...………..….….........................................36

**Figure S33:** ^1^H-NMR spectrum of compound 6 (500 MHz, CDCl_3_**)**…………………………………….......37

**Figure S34:** ^13^C-NMR spectra of compound 6 **(**125 MHz, CDCl_3_**)**…….….….……...………..….…............38

**Figure S35:** DEPT135 of compound 6…………………..………………....…...............................................39

**Figure S36:** COSY of compound 6………………….….….……....………..….….........................................40

**Figure S37:** HMPC of compound 6……………………………………....……………………………….......41

**Figure S38:** HSQC of compound 6………………….….….……...………..….…...........................................42

**Figure S39:** ^1^H-NMR spectrum of compound 7 (500 MHz, C_5_D_5_N**)**…………………………………….........43

**Figure S40:** ^13^C-NMR spectra of compound 7 **(**125 MHz, C_5_D_5_N**)**…….….….……...………..….…...............44

**Figure S41:** DEPT135 of compound 7…………………..………………....…..................................................45

**Figure S42:** COSY of compound 7………………….….….……....………..….…...........................................46

**Figure S43:** HMPC of compound 7……………………………………....…....................................................47

**Figure S44:** HSQC of compound 7………………….….….……...………..….…............................................48

**Figure S45:** ROESY of compound 7…………………………………………………………………………...49

**Figure S1:** ^1^H-NMR spectrum of compound 1 (500 MHz, C_5_D_5_N**)**

**Figure S2:** ^13^C-NMR spectra of compound 1 **(**125 MHz, C_5_D_5_N**)**

**Figure S3:** DEPT135 of compound 1

**Figure S4:** COSY of compound 1

**Figure S5:** HMPC of compound 1

**Figure S6:** HSQC of compound 1

**
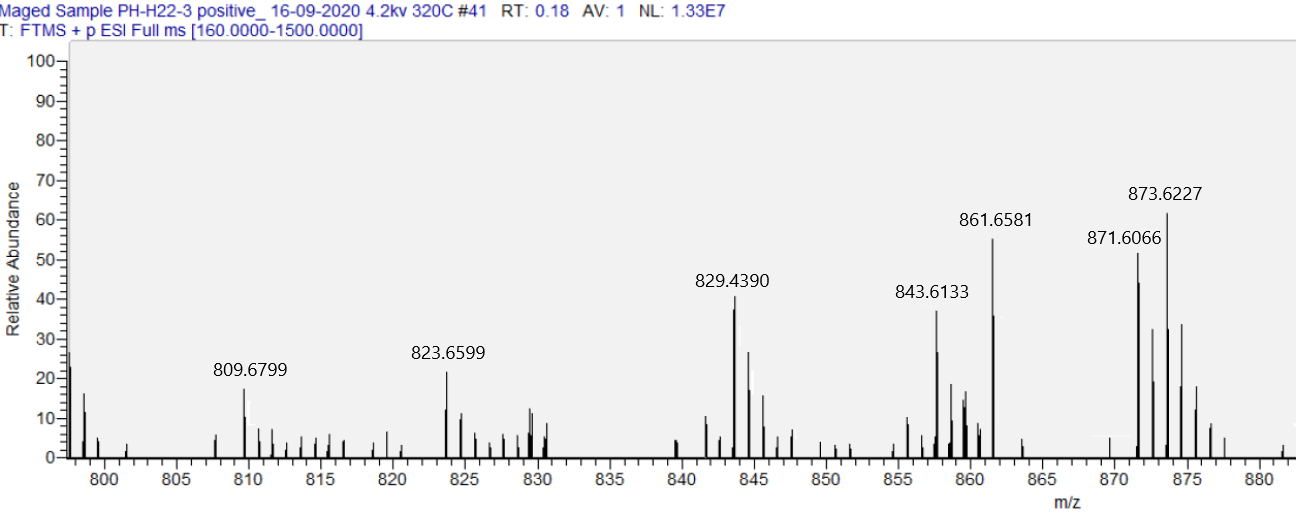
**

**Figure S7:** LC-HRMS analysis (positive mode) [M+Na] + of compound 1

**Figure S8:** GC-MS of the fatty acid liberated from compound 1 after hydrolysis

**Figure S9:** ^1^H-NMR spectrum of compound 2 (500 MHz, CDCl_3_**)**

**Figure S10:** ^13^C-NMR spectra of compound 2 **(**125 MHz, CDCl_3_**)**

**Figure S11:** DEPT135 of compound 2

**Figure S12:** COSY of compound 2

**Figure S13:** HMPC of compound 2

**Figure S14:** HSQC of compound 2

**Figure S15:** ^1^H-NMR spectrum of compound 3 (500 MHz, CDCl_3_**)**

**Figure S16:** ^13^C-NMR spectra of compound 3 **(**125 MHz, CDCl_3_**)**

**Figure S17:** DEPT135 of compound 3

**Figure S18:** COSY of compound 3

**Figure S19:** HMPC of compound 3

**Figure S20:** HSQC of compound 3

**Figure S21:** ^1^H-NMR spectrum of compound 4 (500 MHz, CDCl_3_**)**

**Figure S22:** ^13^C-NMR spectra of compound 4 **(**125 MHz, CDCl_3_**)**

**Figure S23:** DEPT135 of compound 4

**Figure S24:** COSY of compound 4

**Figure S25:** HMPC of compound 4

**Figure S26:** HSQC of compound 4

**Figure S27:** ^1^H-NMR spectrum of compound 5 (500 MHz, CDCl_3_**)**

**Figure S28:** ^13^C-NMR spectra of compound 5 **(**125 MHz, CDCl_3_**)**

**Figure S29:** DEPT135 of compound 5

**Figure S30:** COSY of compound 5

**Figure S31:** HMPC of compound 5

**Figure S32:** HSQC of compound 5

**Figure S33:** ^1^H-NMR spectrum of compound 6 (500 MHz, CDCl_3_**)**

**Figure S34:** ^13^C-NMR spectra of compound 6 **(**125 MHz, CDCl_3_**)**

**Figure S35:** DEPT135 of compound 6

**Figure S36:** COSY of compound 6

**Figure S37:** HMPC of compound 6

**Figure S38:** HSQC of compound 6

**Figure S39:** ^1^H-NMR spectrum of compound 7 (500 MHz, C_5_D_5_N**)**

**Figure S40:** ^13^C-NMR spectra of compound 7 **(**125 MHz, C_5_D_5_N**)**

**Figure S41:** DEPT135 of compound 7

**Figure S42:** COSY of compound 7

**Figure S43:** HMPC of compound 7

**Figure S44:** HSQC of compound 7

**Figure S45:** ROESY of compound 7
