# Supplementary material for: Cytotoxic, Apoptosis-Inducing Activities, and Molecular Docking of a New Sterol from Bamboo Shoot Skin Phyllostachys heterocycla var. pubescens
Source: Molecules. 2020 Nov 30;25(23):5650. doi: 10.3390/molecules25235650 (PMC7731115; doi:10.3390/molecules25235650)
Supplement: Supplementary file 1 [file molecules-25-05650-s001.zip › Electronic supplementry Material (ESM)/Table S1 (Primers).docx]

Primer used were listed in **Table S1**

| **Primer** | **Sequence** |
| --- | --- |
| **β-Actin** | FOR: 5`-GCACTCTTCCAGCCTTCCTTCC-3`  REV: 5`-GAGCCGCCGATCCACACG-3` |
| **P53** | FOR: 5`-CTTTGAGGTGCGTGTTTGTG-3`  REV: 5`-GTGGTTTCTTCTTTGGCTGG-3` |
| **Bcl-2** | FOR: 5`-GAGGATTGTGGCCTTCTTTG-3`  REV: 5`-ACAGTTCCACAAAGGCATCC-3` |
| **BAX** | FOR: 5`-TTTGCTTCAGGGTTTCATCC-3`  REV: 5`-CAGTTGAAGTTGCCGTCAGA-3` |
| **Casp-3** | FOR: 5'- TGGCCCTGAAATACGAAGTC-3'  REV: 5'- GGCAGTAGTCGACTCTGAAG -3' |
| **Casp-8** | FOR: 5'- AATGTTGGAGGAAAGCAAT -3'  REV: 5'- CATAGTCGTTGATTATCTTCAGC -3' |
| **Casp-9** | FOR: 5'- CGAACTAACAGGCAAGCAGC -3'  REV: 5'- ACCTCACCAAATCCTCCAGAAC -3' |

**Table (S1**):- Primers used for real-time RT-PCR
