# Supplementary figures and images for: Cytotoxic, Apoptosis-Inducing Activities, and Molecular Docking of a New Sterol from Bamboo Shoot Skin Phyllostachys heterocycla var. pubescens
Source: Molecules. 2020 Nov 30;25(23):5650. doi: 10.3390/molecules25235650 (PMC7731115; doi:10.3390/molecules25235650)

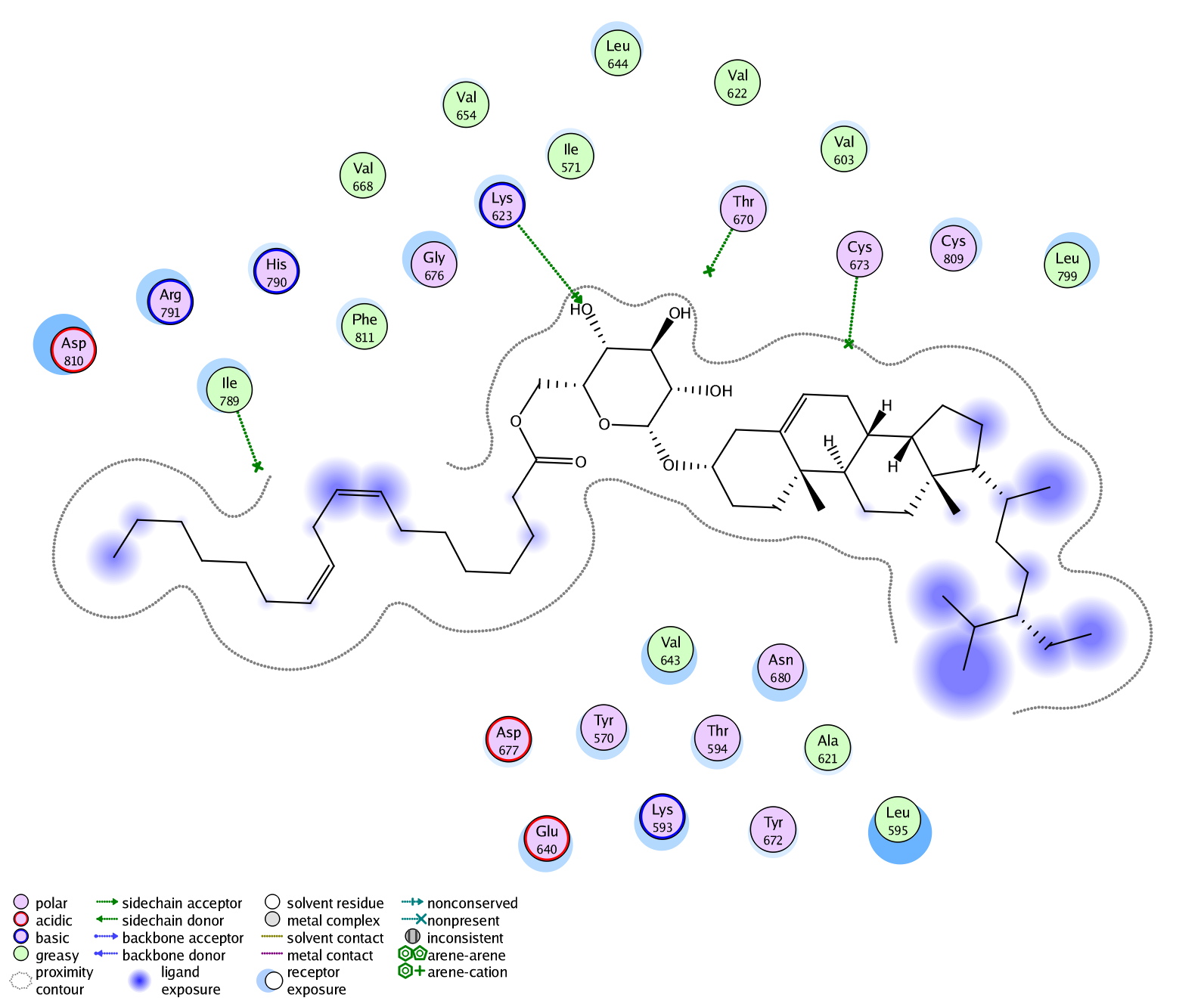

Supplement: Supplementary file 1 [file molecules-25-05650-s001.zip › Electronic supplementry Material (ESM)/Docking/1T46/ph-22-3.bmp]

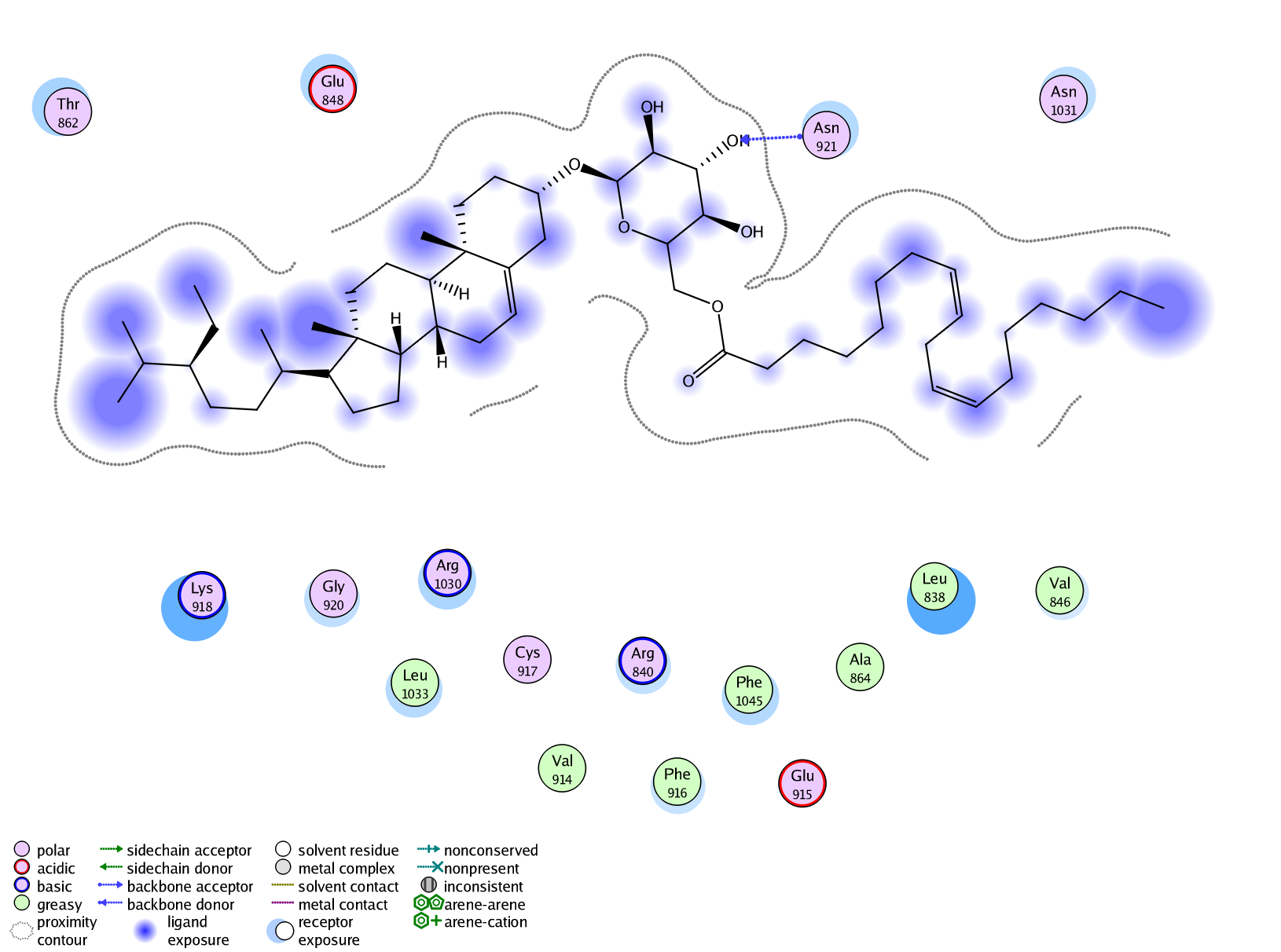

Supplement: Supplementary file 1 [file molecules-25-05650-s001.zip › Electronic supplementry Material (ESM)/Docking/1y6A/ph-22-3.bmp]
