# Supplementary material for: Cytotoxic, Apoptosis-Inducing Activities, and Molecular Docking of a New Sterol from Bamboo Shoot Skin Phyllostachys heterocycla var. pubescens
Source: Molecules. 2020 Nov 30;25(23):5650. doi: 10.3390/molecules25235650 (PMC7731115; doi:10.3390/molecules25235650)
Supplement: Supplementary file 1 [file molecules-25-05650-s001.zip › Electronic supplementry Material (ESM)/CCA report-12092020-(Flow cytometric analysis).doc]

**Researcher**	: Ahmed Khidr elsayed	email: [Ahmedkhider1993@gmail.com](mailto:Ahmedkhider1993@gmail.com)	**mob.** 01066602084

Assay	: Cell Cycle Analysis

Samples	: 02 samples

cell line	: ---

Ref.	: ---

Date	: 12-09-2020

Reader	: BD FACSCalibur

Kit used	: ab139418_Propidium Iodide Flow Cytometry Kit/BD

Solvent	: DMSO

Assay samples	: Cell culture

**Lab Report-I**

| **ser** | Sample data | | Results  DNA content | | | | |
| --- | --- | --- | --- | --- | --- | --- | --- |
|  | **code** | **IC50**  **uM** | %G0-G1 | %S | %G2/M | %Pre-G1 | Comment |
| 1 | PH-20-2/MCF7 |  | 44.15 | 17.36 | 38.49 | 38.21 | cell growth arrest@ S |
| 2 | PH-22-3/MCF7 |  | 48.31 | 24.95 | 26.74 | 25.94 | cell growth arrest@ G2/M |
| 3 | cont.MCF7 |  | 53.28 | 39.26 | 7.46 | 1.37 | --- |

| **s** | **code** | **conc** | **Apoptosis** | | | **Necrosis** |
| --- | --- | --- | --- | --- | --- | --- |
|  |  |  | Total | Early | Late |  |
| 1 | PH-20-2/MCF7 |  | 38.21 | 2.69 | 13.44 | 22.08 |
| 2 | PH-22-3/MCF7 |  | 25.94 | 5.23 | 11.4 | 9.31 |
| 3 | cont.MCF7 |  | 1.37 | 0.36 | 0.15 | 0.86 |

**Detailed results**
